# DOM-mediated membrane retention of fluoroquinolone as revealed by fluorescence quenching properties

**DOI:** 10.1038/s41598-017-05635-z

**Published:** 2017-07-14

**Authors:** Shuang Liang, Li Lu, Fangang Meng

**Affiliations:** 1 Shandong Provincial Key Laboratory of Water Pollution Control and Resource Reuse, School of Environmental Science and Engineering, Shandong University, Jinan, 250100 China; 2 School of Environmental Science and Engineering, Sun Yat-sen University, Guangzhou, 510275 PR China

## Abstract

In this study, membrane filtration tests showed that the membrane rejection degree of difloxacin hydrochloride (DFHC) increased significantly in the presence of Suwannee River DOM or Aldrich humic acid (2–10 mg-C/L). Titration experiments showed that the excitation and emission of Peak R belonging to DFHC exhibited blue shifts by 5 nm and 10 nm, respectively, in the presence of DOM. The presence of DFHC can, in turn, lead to more significant overlapping of the fluorescence peaks of the Suwannee River DOM and Aldrich humic acid. The parallel factor analysis (PARAFAC) of the excitation-emission matrix (EEM) spectra can well decompose the components belonging to DFHC from the DOM + DFHC mixtures. The maximum fluorescence intensity (*FI*_max_) of the antibiotic-like component (C1) sharply decreased upon the initial addition of DOM. More specifically, the Aldrich humic acid showed a larger quenching effect on DFHC than the Suwannee River DOM. The stability constants (*K*_*M*_) obtained by the Ryan and Weber model also corroborated that the Aldrich humic acid had a much higher binding stability (*K*_M_ = 4.07 L/mg) than the Suwannee River DOM (*K*_M_ = 0.86 L/mg). These results have great implications for our understanding of the membrane filtration behavior of trace contaminants in natural waters.

## Introduction

Membrane processes have been increasingly employed in wastewater and water treatment to remove particles, dissolved organic matter (DOM) and micropollutants because of their technical advantages (e.g., relatively effective treatment, low footprint requirements) compared with conventional treatment systems^1, 2^. A considerable number of previous studies have demonstrated the efficient removal of typical micropollutants, such as PPCPs, EDCs and antibiotics, by NF and UF membranes^3–5^. In addition to size exclusion by the membrane separation, interaction mechanisms such as electrostatic repulsion^6^ and hydrophobic adsorption^7^ were reported to be responsible for the removal of these micropollutants by membranes^3^. Specifically, steric hindrance can largely influence the rejection of uncharged trace organics, while the rejection of polar trace organic mainly depends on the electrostatic interactions under the situation of a charged membrane surface^8^. The hydrophobic adsorption, molecular size and compound hydrophobicity were crucially correlated with the removal of pesticides by membranes^9^. The pH and ionic strength were also recognized to be important factors affecting the rejection of emerging micropollutants^10, 11^. This is likely because the surface charge of the membranes and the dissociation of the polar organics could be affected by the pH value, e.g., the membrane rejection (10–70%) of PPCP varied significantly with the pH variation (pH = 4–10) during the microfiltration^11^. In addition, the presence of humic acid was observed to play a more important role in regulating the rejection degrees of pesticides than did inorganic salts^12^.

DOM, which ubiquitously occurs in sediment and water ecosystems, is a widespread complexing agent^13^. The hydrophobic humic acids make up the dominant fraction of DOM in natural water and are generally employed as the model compound for DOM. The humic acids can combine with the membrane during water treatment using a membrane process^14, 15^. Numerous studies have revealed that the high content of oxygen-containing functional groups such as phenolic, alcoholic and carboxylic groups in the DOM imbues it with high complexation capacities for contaminants^16–19^. As such, the fate of the contaminants can be potentially changed by the formation of contaminant-DOM complexes^16^. As previously hypothesized, physico-chemical properties including charge and functionality affect the binding between DOM and PPCPs^16^. Thus, the binding between the DOM and antibiotics was recognized to be impacted by both hydrophobic effects (e.g., cation bridge and hydrogen bonding) and the intermolecular mechanism of association^20^.

Humic substances (HS), as the major fraction of DOM in natural water, are expected to play primary roles in binding with antibiotics of both hydrophilic and hydrophobic moieties owing to H-bonding and other intermolecular interactions^17^. It has been shown that ciprofloxacin can bind with HS due to intermolecular interactions, and both the pH and the types of HS determine the ciprofloxacin-HS complexes^17^. Of note, under environmentally relevant pH conditions, the primary driver for ciprofloxacin adsorption onto soil organic matter is a cation-exchange reaction involving a mediating electrostatic attraction between the positively charged group of the antibiotic and the negatively charged HS group^17, 21^. In fact, the binding of ciprofloxacin with HS mainly depends on the van der Waals interaction, H-bonding and electrostatic interactions^17^. In all, the binding interactions between antibiotics and HS are expected to be of high significance for the membrane rejection of antibiotics. However, few studies have examined the mechanisms underlying the influence of DOM or HS on the membrane retention of PPCPs. Such a study would aid in understanding the behavior of PPCPs in both natural water and engineered systems.

Fluoroquinolone antibiotics (FQs) are a type of broad-spectrum antibacterial agent commonly used in human and veterinary medicine^22–24^. FQs have been widely detected in soil and water ecosystems^25, 26^. Reports of FQs in surface waters and wastewater effluents employed as supplies of drinking water have attracted increasing attention from the public^26, 27^. Difloxacin hydrochloride (DFHC) was selected as a proxy of the FQs because of its potential risks to the environment^24^. This study sought to reveal the correlation between the retention degree during UF and the fluorescence quenching efficiency of DFHC in the presence of DOM. We employed fluorescence quenching spectroscopy to elucidate the interaction of DOM with DFHC under environmentally relevant pH and temperature conditions. Our study appears to be the first to reveal the role(s) of DOM in changing DFHC through fluorescence quenching.

## Materials and Methods

### Experiment materials

All chemicals used in this study were ACS reagent grade unless otherwise noted. The Suwannee River DOM (SRDOM, 2R101N) and Aldrich humic acid (HA) were purchased from the International Humic Substance Society (IHSS) and the Aldrich Chemical Company, respectively. Difloxacin hydrochloride (DFHC) was purchased from Langchem Corp. (China). DOM (i.e., SRDOM and HA) and DFHC stock solution were prepared by dissolving the respective powders in ultrapure water (18.2 MΩ cm) and then filtering through glass fiber membranes with a pore size of 0.45 *μ*m. The concentrations of the SRDOM and HA stock solutions were 100 mg-C/L (as dissolved organic carbon (DOC)), which was determined using a TOC analyzer (TOC-L CPH, Shimadzu, China). The DFHC stock solution was prepared with a concentration of 4 mg/L. These organic solutions were stored under refrigeration (4 °C) until needed. NaCl solution was prepared to adjust the background ion strength of the water samples to 0.01 M, and the pH of the water samples was adjusted to approximately 7.0 using 0.1 M HCl or NaOH solution.

### Ultrafiltration experiment

Dead-end filtration tests were conducted with commercially available PES ultrafiltration (UF) membranes (Microdyn-Nadir Corp., Germany) with molecular weight cut-offs of 20 and 50 kDa in a 300-mL stirred cell (MSC 300, Mosu Corp., Shanghai, China). A fresh UF membrane was used for each filtration test, and the membranes were soaked in ultrapure water for at least 24 h to remove impurities. The UF system was driven by an 80-kPa transmembrane pressure obtained from a N_2_ gas cylinder controlled by a pressure regulator (Type 10, Bellofram Corp., USA), and the stirring speed of the cell was set to 300 rpm. Before the feed solution filtration, 100 mL of ultrapure water was passed through the UF membrane for stabilization. The UF tests were performed with 0, 2, 4, 6, 8 and 10 mg-C/L SRDOM, HA or SRDOM-HA (with a ratio of SRDOM to HA of 1:1) in the feed solution with the DFHC concentration fixed at 300 *μ*g/L. The prepared solution was shaken for 12 h to ensure full interaction between the DOM and DFHC. A filtrate flow rate of 300 mL feed solution was controlled using an analytical balance connected to a computer, and filtrate samples were collected for TOC, UV_254_, fluorescence and HPLC analyses. At the end of each filtration test, the stirred cell was cleaned with ultrapure water and acids to remove the residual substances.

In this study, the DOM-DFHC solution was also subsequently filtered through a series of membranes with different molecular weight cut-offs (PVDF material, 100 kDa, 10 kDa, 3 kDa) to understand the capacity of each fraction of DOM to bind DFHC.

### Detection of DFHC using HPLC with an ultraviolet detector

The removal of DFHC in the filtration experiments was analyzed by a high-performance liquid chromatograph (HPLC, Ultimate 3000, USA) equipped with a C18 column (Poroshell 120, EC-C18, 4.6 × 50 mm, 2.7 *μ*m) and an ultraviolet detector at room temperature (25 °C) with a detection wavelength of 280 nm. The mobile phase was a mixture of acetonitrile and 0.1% formic acid (V/V, 20/80) under isocratic conditions at a flow rate of 0.3 mL/min. The injection volume of each sample was 20 *μ*L, and the retention time for DFHC was in the range of 5.0–6.0 min. The rejection degree (*R*) of DFHC by the UF membranes was calculated using Eq. (1),

1 $${\rm{R}}( \% )=\frac{{C}_{f}-{C}_{p}}{{C}_{f}}\times 100 \% $$

where *C*_*f*_ is the feed concentration (*μ*g/L) and *C*_p_ is the permeate concentration (*μ*g/L).

### Fluorescence quenching experiment

A series of 10-mL brown bottles cleaned with ultrapure water and dried thoroughly at 105 °C were used for the fluorescence quenching experiments. The fluorescence quenching of DFHC with the end-members of DOM (SRDOM and HA) or their mixture (SRDOM + HA with a ratio of 1:1) was conducted as follows. 5-mL aliquots of a dilute solution of DOM were titrated into 10-mL vials containing DFHC. The DOM concentration was in the range of 0–12 mg-C/L (as dissolved organic carbon (DOC)) in the final solution, and the final concentration of DFHC in the final solution was fixed at 300 *μ*g/L. In a similar way, the fluorescence titration of DOM with a fixed concentration of 6 mg-C/L by DFHC varying in the range of 0–320 *μ*g/L was performed. The pH of all the solutions was controlled at approximately 7.0 using HCl or NaOH, and the titrated solution was shaken for 2 h at room temperature (25 °C) in the dark for full complexation. Each titration experiment was performed in triplicate.

### EEM measurements and PARAFAC analysis

To elucidate the interaction between DFHC and DOM, we measured the three-dimensional excitation-emission matrix (3D-EEM) of all model solutions based on the method described by Meng *et al*.^28^. The EEM spectra of the water samples were obtained using a fluorescence spectrometer (F-4500, Hitachi, Japan) equipped with a xenon lamp as a light source. The EEM spectra were collected every 5 nm over excitation wavelengths ranging from 235 to 400 nm at an emission range of 270–550 nm with a 3.36-nm increment. The slit size was set to 5 nm, and the scan speed was controlled at 1200 nm/min. A 290-nm emission cutoff filter was used to eliminate the Raleigh light scattering. The sample EEM was achieved by subtracting the EEM of ultrapure water and measuring in triplicates for reliability^29^. The measurements of the EEM spectra were conducted at room temperature (25 E).The PARAFAC model used for the EEM spectra analysis was constructed with the aid of MATLAB 8.0 using the “N-way Toolbox” and “DOMFluor Toolbox”. The PARAFAC model was employed to analyze a total of 367 EEM datasets for water samples. To determine the appropriate number of components, random initialization and split-half validation were conducted^29^. The observed quenching efficiency (Q) of the DFHC component was calculated using Eq. (2),

2 $${\rm{Q}}( \% )=\frac{{F}_{i}-{F}_{a}}{{F}_{i}}\times 100 \% $$

where *F*_*i*_ is the initial fluorescence intensity of DFHC (RU) and *F*_*a*_ is the fluorescence intensity of DFHC after quenching (RU).

### Complexation

The interaction between the DOM components and DFHC was also evaluated using the complexation model proposed previously^19, 30^. The assumption that the binding between DOM and DFHC occurred at identical and independent sites with 1:1 stoichiometry was considered as a prerequisite for this model, as was the assumption that a linear relationship exists between the ligand concentration and the quenched DFHC fluorescence intensity. Compared to the direct “peak-picking” fluorescence intensity, the independent components extracted from the PARAFAC analysis were more suitable for this model^31, 32^. The complexation parameters used to describe the binding potentials were obtained by nonlinear fitting using Eq. (3),

3 $$\begin{array}{rcl}{\rm{I}} & = & {I}_{0}+({I}_{ML}-{I}_{0})(\frac{1}{2{K}_{M}{C}_{L}})((1+{K}_{M}{C}_{L}+{K}_{M}{C}_{M})\\  &  & -\sqrt{{(1+{K}_{M}{C}_{L}+{K}_{M}{C}_{M})}^{2}-4{K}_{M}^{2}{C}_{L}{C}_{M}})\end{array}$$

where *I* and *I*_0_ are the fluorescence intensity (*FI*_*max*_) with and without the quencher, respectively; *I*_*ML*_ is the limiting value below which enhancing the dosage of the quencher has no influence on the fluorescence intensity; *C*_*L*_ is the total ligand concentration; and *K*_*M*_ is the conditional stability constant. *I*_*ML*_, *K*_*M*_ and *C*_*L*_ were calculated using the Levenberg-Marquardt and Universal Global Optimization (LM-UGO) method of 1stOpt software. Based on the Ryan-Weber model, the parameter number can be reduced to support the approach described previously^33^. A constant value for *I*_*ML*_/*I*_0_ can be achieved by the equation

4 $$|\frac{I}{{I}_{0}}-1|=|\frac{{I}_{ML}}{{I}_{0}}-1|(1-{e}^{-\alpha {C}_{M}})$$

where $(\frac{{I}_{ML}}{{I}_{0}}-1)$ and α are the fitting parameters. It is possible to assess the parameters *K*_*M*_ and *C*_*L*_ by further modification. Moreover, the fraction of the initial fluorescence corresponding to the binding of fluorophores (*f*) was determined using Eq. (5):

5 $$f=\frac{({I}_{0}-{I}_{ML})}{{I}_{0}}$$

### Statistical analyses

In this study, regression and correlation analysis obtained from SPSS 20.0 software were employed to characterize the relationship between the fluorescence quenching efficiency and membrane rejection degree. The significance levels were recorded as non-significant (*p* > 0.05), significant (0.01 < *p < *0.05) and highly significant (*p* < 0.01).

## Results and Discussion

### DOM-mediated membrane rejection of DFHC

**Figure 1:**
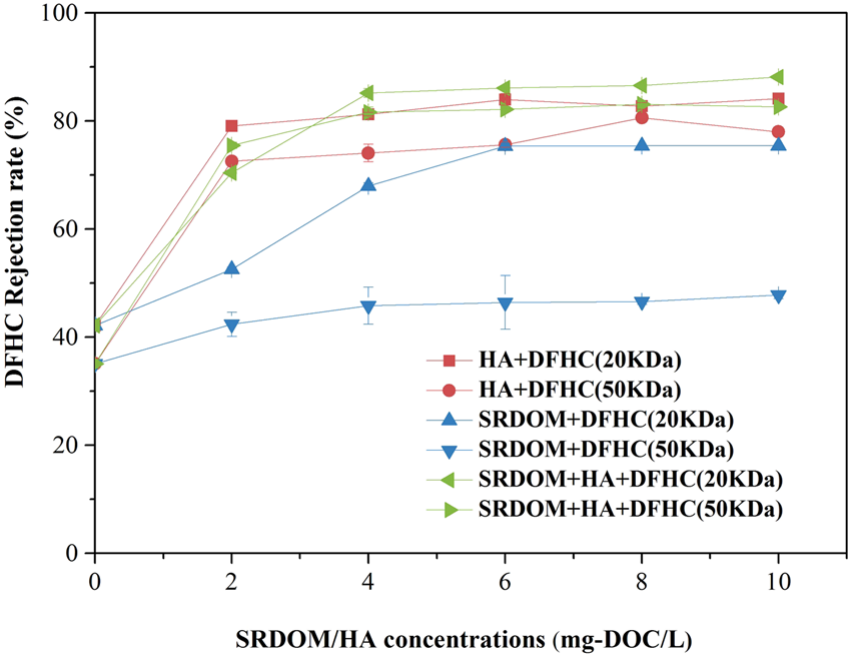
Rejection degrees of DFHC by ultrafiltration membranes (20 kDa and 50 kDa) in the presence of SRDOM, HA and their mixture at pH = 7.0.

As seen in Fig. 1, the membrane rejection degrees of DFHC by two membranes with pore sizes of 20 kDa and 50 kDa in the absence of DOM were determined to be 42% and 35%, respectively. This implies that the UF membranes used in this study have a lower capability to reject the DFHC antibiotic. This is consistent with previous reports that PPCPs from water cannot be well-rejected by UF membranes^4, 5^. As the two UF membranes are of the same material and surface properties, besides their different pore sizes, the increased rejection degrees by the 20-kDa membranes over the 50-kDa ones were attributed to size exclusion. This is in good agreement with previous findings^34, 35^. In comparison with the control DFHC solution, the presence of SRDOM or HA significantly increased the rejection degree of DFHC, which increased to 60–88% for most filtrate solutions. One exception is that the presence of SRDOM alone showed only minor effects on the improvement of the DFHC retention during the UF using 50-kDa membranes. There could be two reasons explaining this phenomenon: (i) low interaction potentials between SRDOM and DFHC and (ii) a smaller molecular size of SRDOM. In this study, we found that 50% of the HA molecules (or molecule aggregates) used here were of a size larger than 100 kDa (Supplementary materials, Table S1), much higher than that for the SRDOM (<1%). Previous studies also showed that SRDOM has an average molecular weight of 2644 Da^16^, while that of HA is mainly distributed in the range of 30 kDa–0.22 *μ*m^36^. The HA used in both our study and previous study originated from soil, which is of large size than that from aquatic systems. As such, the HA (76% for 20 kDa and 69% for 50 kDa on average) exhibited a higher rejection degree than did SRDOM (65% for 20 kDa and 44% for 50 kDa on average). The interactions between SRNOM or HA and DFHC will be presented in the following sections. Strikingly, the mixture of SRDOM and HA showed a more significant role in improving the DFHC retention than its end members (i.e., SRDOM alone and HA alone), which is attributable to the formation of macromolecular components resulting from the inter-component interactions between SRDOM and HA^37^.

### Fluorescence landscape of DFHC, DOM and their mixtures

**Figure 2:**
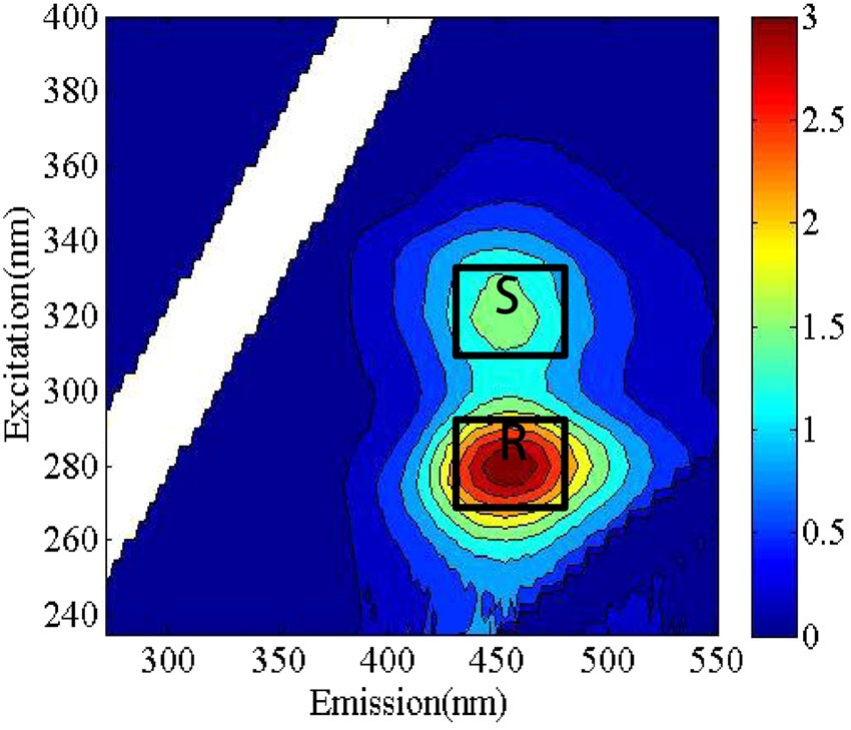
EEM spectra of DFHC (300 *μ*g/L).

**Figure 3:**
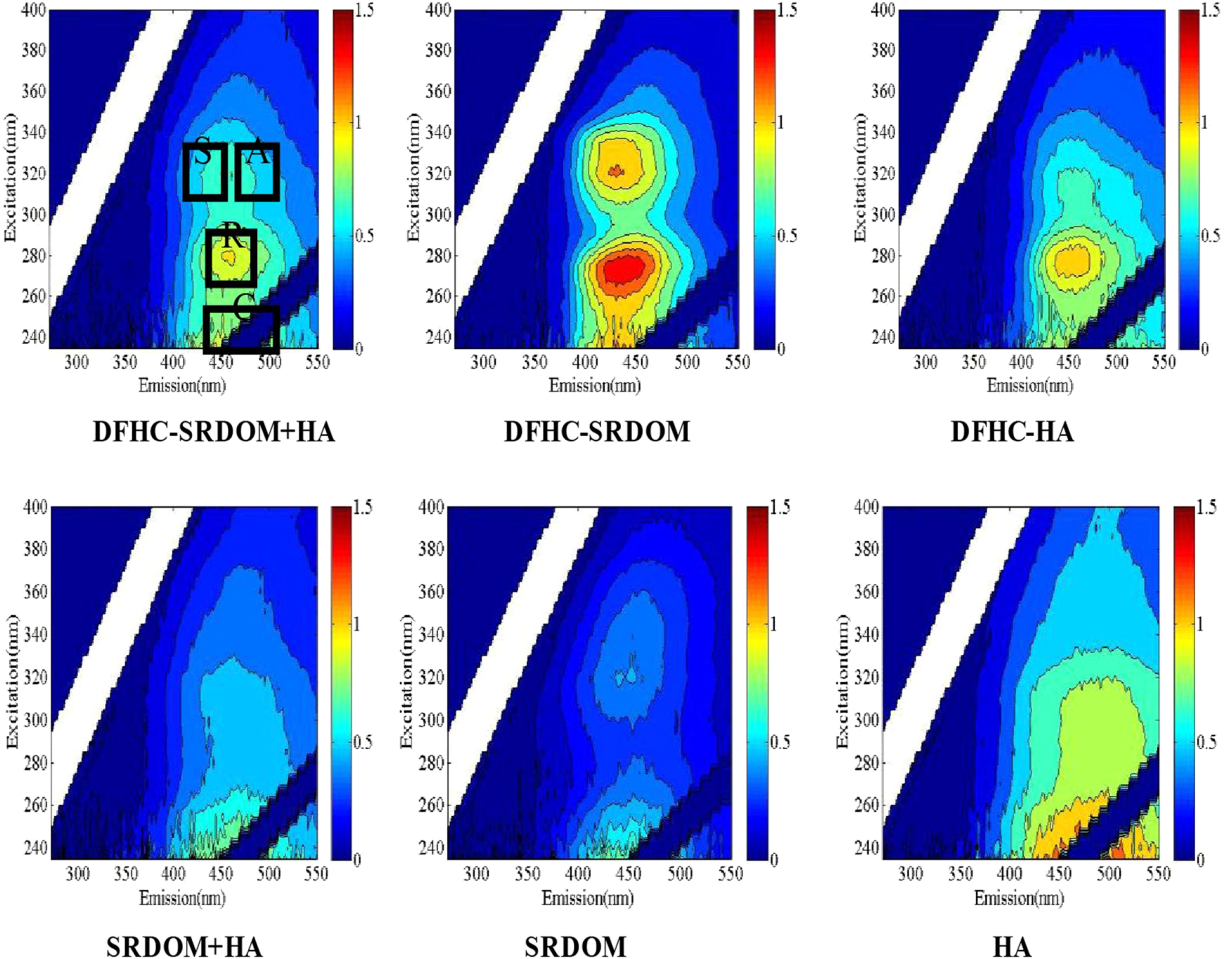
EEM spectra of SRDOM, HA and four mixtures. The concentrations of DFHC, SRDOM and HA are 300 *μ*g/L, 6 mg/L and 6 mg/L, respectively.

As presented in Fig. 2 and Fig. 3, the EEM spectrum of the DFHC solution was characterized by two peaks at Ex/Em = 280/450 nm (peak R) and Ex/Em = 320/450 nm (peak S). The SRDOM solution showed two significant peaks at Ex/Em = 240/450 nm (peak C) and Ex/Em = 320/450 nm (peak A), which was thought to be indicative of the presence of terrestrial humic-like substances^18^. HA showed two EEM peaks at Ex/Em = <250/490 nm (peak C) and Ex/Em = 290/490 nm (peak A). Normally, peak C is attributable to the presence of an aquatic humic substance, as reported previously^38^. Both SRDOM and HA had no fluorescence peaks in the low Ex/Em region of their EEM spectra, indicating no presence of protein-like components. In fact, the DOM end-member (e.g., SRDOM or HA) exhibited an overlap phenomenon between the two main peaks, suggesting the occurrence of inter-component interactions within the DOM components. More strikingly, the EEM spectrum of the SRDOM-HA mixture was largely different from those of their end-members, for instance, there was a relative blue shift (10 nm) in the excitation and a red shift (10 nm) in the emission of Peak A occurred compared to that of the end member of SRDOM (Fig. 3(e)). In addition, peak A of SRDOM-HA exhibited blue shifts by ca. 10 nm and 30 nm in the excitation and emission wavelengths, respectively, compared with those in the end-member of HA.

Furthermore, the inclusion of DFHC in the DOM (that is, SRDOM, HA, or their mixture) also led to significant overlapping phenomena. The excitation and emission of Peak R belonging to DFHC exhibited blue shifts by 5 nm and 10 nm, respectively, in the presence of DOM. The emission of Peak A belonging to the HA also shifted by 20 nm due to the presence of DFHC. Likewise, the emission of peak C belonging to the end-member of SRDOM exhibited a blue shift by 10 nm in the presence of DFHC. Furthermore, Peak A of the EEM spectrum of the SRDOM-DFHC mixture almost completely overlapped with peak S, implying that the peak-picking methods cannot well differentiate between peaks A and S in the spectrum of the SRDOM-DFHC mixture. More specifically, Peak R elongated distinctly into Peaks C in the EEM spectra of the DFHC-SRDOM, DFHC-HA and DFHC-SRDOM-HA mixtures. These results indicate the DFHC took place a complex interaction with DOM components. It has been documented that inter-molecular energy transfer and the existence of multiple fluorophores both always give rise to the overlap of EEM peaks^39^. As some of these end-members yielded fluorescence peaks in similar regions, the presence of multiple fluorophores is a potential reason for the occurrence of overlapping peaks^40, 41^. Nevertheless, we cannot rule out the possibility of inter-molecular energy transfer. Previous studies implied that multiple mechanisms including electrostatic interactions and hydrogen bonding may lead to the interaction of fluoroquinolone-DOM^17, 42^. The investigation of the interaction between antibiotic-like fluorophores (e.g., Peaks R and S) and the fluorophores in Peaks A and C is necessary for further understanding the behavior of PPCPs in natural or engineered systems.

### PARAFAC modeling of DFHC-DOM Interactions

**Figure 4:**
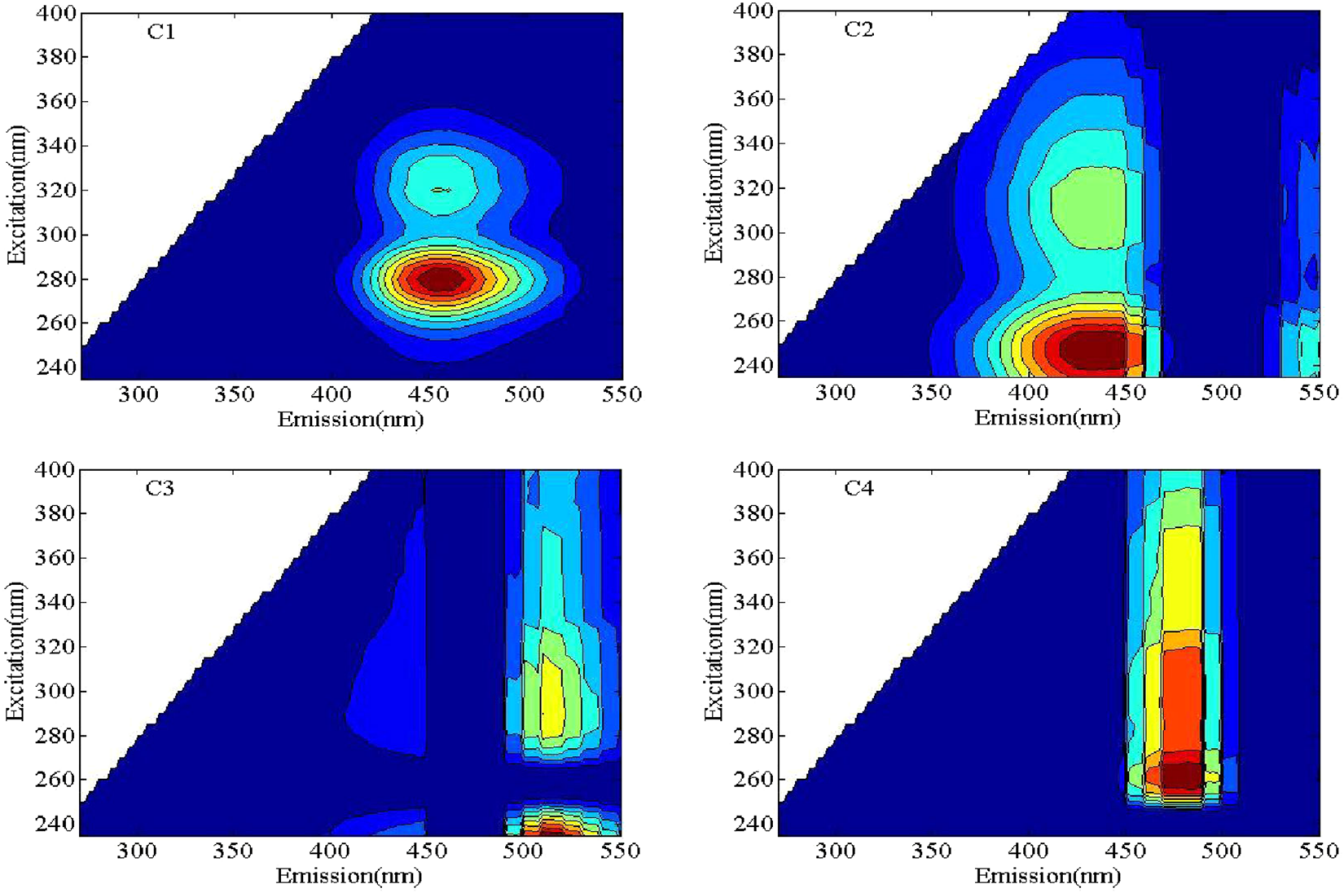
EEM contour plots of the four components identified by the DOM Fluor-PARAFAC model.

The peak picking methods cannot well evaluate the exact changes in the fluorescence intensities of the mixtures described above because of the overlapping among some peaks in the spectra. In comparison, PARAFAC modeling can aid in extracting and separating the overlapping EEM peaks. Therefore, in this study, the PARAFAC modeling of 367 EEM datasets was conducted based on the tutorial^43^. The PARAFAC modeling successfully decomposed the EEM spectra into four independent components with a validation >90% (required value >80%)^44^. It can be seen that the position and shape of component 1 (C1, Fig. 4a) were really the same as those of the EEM peaks of the DFHC end-member shown in Fig. 4a. The other three components (C2, C3 and C4) were attributed to humic-like components (Table 1). More detailed analysis also shows that C1 only occurred in the DFHC-containing samples, while C2, C3 and C4 appeared in the HA- or SRDOM-containing samples. These results suggest that the component belonging to DFHC in the mixtures was well extracted from the EEM spectra of the mixtures using the PARAFAC model.

**Table 1:** Characteristics of four components identified in this study in comparison with those identified in previous reports.

Component	Ex/Em wavelength (nm)	Description and source assignment	Occurrence references
C1	280 (320)/450	Antibiotic-like	
C2	250 (315)/430	Humic-like	53
C3	290 (<250)/510	Humic-like	53, 54
C4	260 (290) (370)/480	Humic-like	53

**Figure 5:**
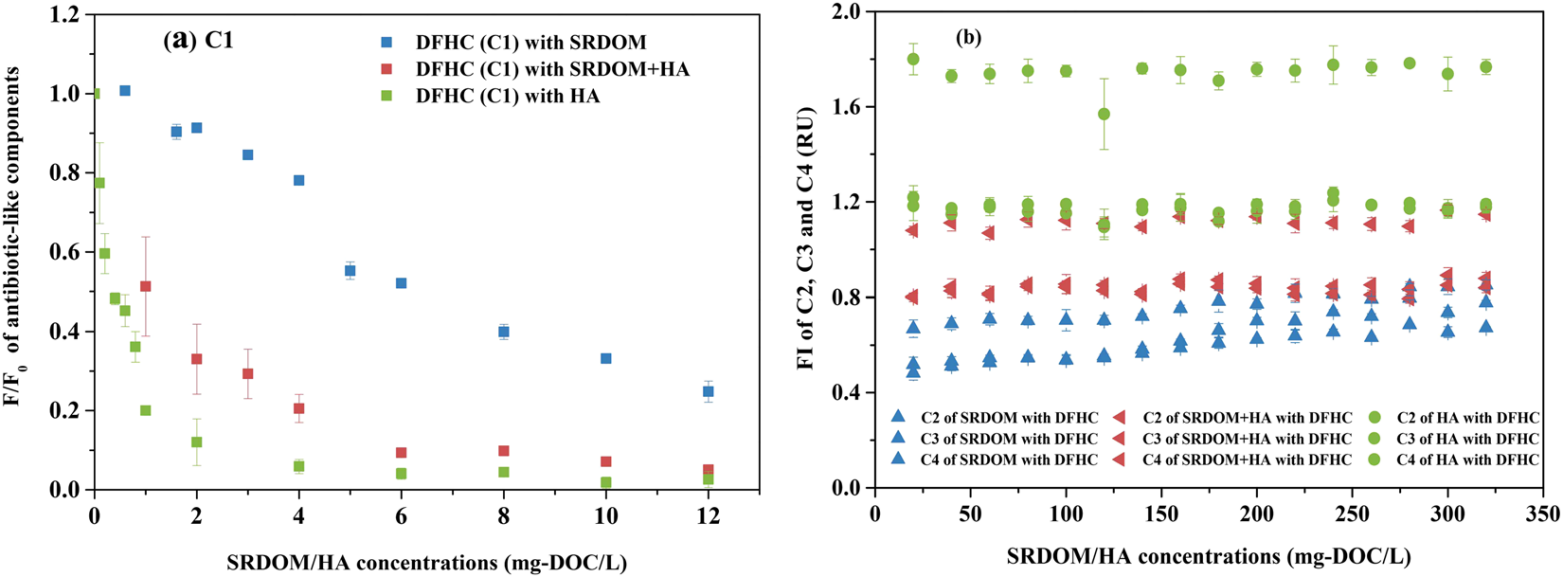
Changes in fluorescence intensity during the fluorescence titration. (**a**) Changes in fluorescence intensity of the antibiotic (C1) at a concentration of 300 *μ*g/L dosed with SRDOM, SRDOM + HA and HA(0–12 mg-DOC/L), where F_0_ is the fluorescence intensity of DFHC at 300 *μ*g/L in the absence of SRDOM and HA, (**b**) Changes in fluorescence intensity of three humic-like components belonging to SRDOM and HA at a concentration of 6 mg-DOC/L when dosed with antibiotic (0–320 *μ*g/L).

Representative fluorescence-quenching curves of the antibiotic-like component with the addition of the SRDOM, HA and SRDOM-HA mixture are shown in Fig. 5(a). The fluorescence quenching was presented as percent changes from initial levels (*F*_max_/*F*_max0_, where *F*_max_ is the fluorescence intensity of each sample and *F*_max0_ is the fluorescence intensity of DFHC without quencher). Therefore, the fluorescence quenching curves reflected the changes in the maximum fluorescence intensity (*FI*_max_) of the antibiotic-like component with the increasing concentration of DOM. It can be seen that the *FI*_max_ of the antibiotic-like component (C1) sharply decreased upon the initial addition of SRDOM, SRDOM + HA or HA, implying that the presence of humic-like components (e.g., SRDOM or HA) could give rise to a significant alteration in the properties of the antibiotic-like component. It can be noted that the HA showed the largest quenching effects on DFHC, followed by SRDOM + HA and SRDOM. These differences are likely due to the higher abundance of carboxylic and phenolic groups, which are the major binding sites for chemicals, in HA in relative with that in SRDOM^45^. Bo *et al*. also reported that the carboxylic groups were most likely the predominant binding sites for metal ions^42^. In our study, the FTIR characterization showed that the HA sample contained much stronger peaks in the range of 1500–1800 cm^−1^, which is attributable to the presence of C = O bonds in amide groups, aromatic substances or quinone substances and/or the presence of the unsaturated C = C stretching and C-O stretching of carboxylic acids, than that of the SRDOM (Supplementary file, Figure S2, Table S2 and Table S3). The XPS analysis also supported that the HA was richer in oxygen-containing compounds than the SRDOM (Supplementary file, Figure S3 and Table S4), which can facilitate the interaction between the metal ions and DOM molecules. However, the exact mechanism of the different binding behaviors of these two varieties of DOM needs to be further explored in a robust manner.

On the basis of the antibiotic-like components quenched by humic-like components, the fluorescence quenching efficiency of C1 was calculated with Eq. (2). As can be seen from Fig. 5a, the fluorescence quenching of C1 by SRDOM + HA and HA increased sharply initially (0–2 mg-DOC/L) and then slowed down, finally being maintained at a relative steady level when the concentrations of SRDOM + HA and HA reached higher levels (>6 mg-DOC/L). In contrast, the fluorescence quenching efficiency of C1 by the SRDOM always increased in the entire DOC range, but with lower values than those by the SRDOM + HA or HA. The SRDOM, SRDOM + HA and HA yielded significant quenching of the antibiotic-like component (C1) of 74%, 94% and 97% at a fixed final DOC concentration of 12 mg/L, respectively (see Fig. 4). These results further indicate that the HA had a higher capability to bind DFHC.

**Figure 6:**
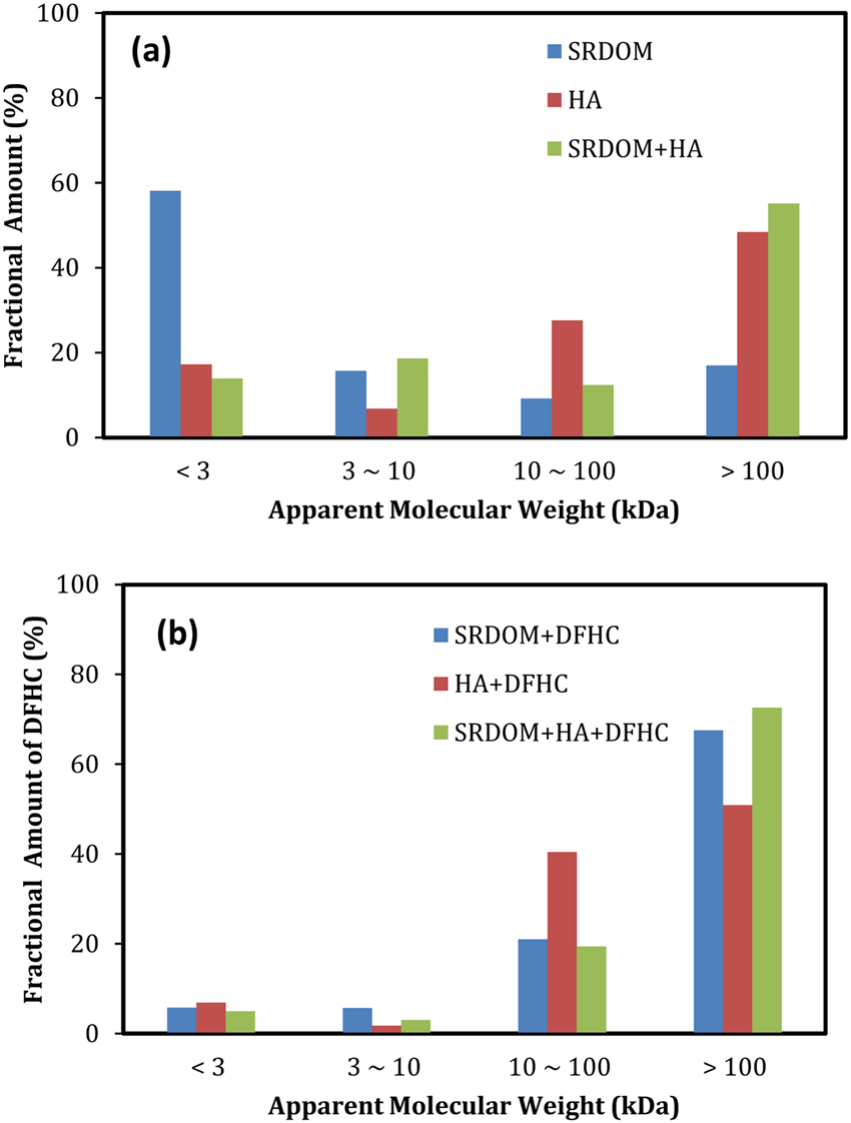
Molecular size distribution of DOM (**a**) and the binding of DFHC on the size-fractionated DOM (**b**).

The fitted stability constants (*K*_*M*_) of SRDOM, SRDOM + HA and HA were calculated using the Ryan and Weber model for the PARAFAC-derived antibiotic-like component (C1) (Table 2). The calculated *K*_M_ values were 0.86, 4.07 and 2.76 L/mg for SRDOM, HA and SRDOM + HA, respectively (Table 2). Therefore, the DFHC had a higher binding potential with the HA and SRDOM + HA mixture compared with that with SRDOM. This can likely be attributed to the higher total ligand concentration of HA compared to SRDOM, as characterized by FTIR and XPS. In all, the DOM source or specific composition was of higher significance for the binding and transport of PPCPs in aquatic environments. More interestingly, we also noted that the DFHC tended to bind with large-size fractions of DOM (>100 kDa and 10–100 kDa) for both SRDOM and HA (Fig. 6(b)). Although SRDOM is of small size, more than 90% of the DFCH was found to bind with large-size SRDOM (>10 kDa). These results also imply that the large-size DOM molecules have great capability to capture PPCPs in aquatic environments.

**Table 2:** Fitting parameters of the complexation model for the data obtained in the titration experiments

	*K*_*M*_(L/mg)	*I*_*ML*_	${\boldsymbol{f}}{\boldsymbol{=}}{\boldsymbol{(}}{\bf{1}}{\boldsymbol{-}}\frac{{{\boldsymbol{I}}}_{{\boldsymbol{M}}{\boldsymbol{L}}}}{{{\boldsymbol{I}}}_{{\bf{0}}}}{\boldsymbol{)}}{\boldsymbol{\times }}{\bf{100}}{\boldsymbol{ \% }}$	*R* ^2^
SRDOM	0.86	0.19	0.84	0.97
SRDOM + HA	2.8	0.24	0.92	0.98
HA	4.07	0.18	0.95	0.98

Note: *K*_*M*_, *I*_*ML*_ and *f* represent the conditional stability constant, limiting fluorescence intensity value below which the FI does not change and fraction of the fluorescence intensity that corresponds to the binding fluorescence, respectively.

In this study, we also found that the interactions between SRDOM-SRDOM, HA-HA and DFHC-DFHC are ignorable, as revealed by the linearly increased *FI*_max_ of each end-member with the increasing concentration (0–12 mg-DOC/L for SRDOM and HA, 0–300 *μ*g/L for DFHC) (see Supplementary Materials, Figure S4(b), S4(c) and S4(d)). More crucially, the fluorescence titration of the humic-like end-members (at a fixed final DOC concentration of 6 mg/L for SRDOM, SRDOM + HA and HA) with the addition of DFHC (with concentrations ranging from 0 to 320 *μ*g/L) showed that the increasing concentration of antibiotic-like components did not result in a fluorescence quenching effect on the humic-like components (see Fig. 5(b)). Moreover, the UV-vis spectra of SRDOM, SRDOM + HA and HA did not change significantly with the addition of DFHC (0–320 *μ*g/L) (see Supplementary Materials, Figure S5). The results above suggest that the antibiotic-like components and humic-like components only exhibited unidirectional interactions rather than bidirectional interactions.

### Implications of this study

In recent years, membrane filtration has been widely employed for drinking water production or wastewater reuse. Although the membrane processes, particularly for low-pressure membranes, are designed with the primary aim in eliminating microbes and DOM, the membranes also contribute largely for the rejection of PPCPs^3^. As the micropollutants are of smaller size than membrane pores (e.g., UF membranes), the rejection by membranes could be due to the interaction of PPCPs with DOM either in cake layer or in water bulk^46, 47^. Nonetheless, there have been rare studies to reveal the role(s) of DOM-PPCPs interactions in regulating the membrane rejection behavior of PPCPs. The co-occurrence of DOM and PPCPs are ubiquitous in aquatic environments. As such, the potential formation of DOM-PPCP complexes would determine their fate and behavior, particularly for PPCPs, in natural waters and engineered systems. The elongation of the EEM spectrum peaks was generally caused by the inter-molecular interactions or the presence of multiple fluorophores, e.g., it has been found that DOM can undergo inter-component interactions and form a supramolecular assembly structure as a result of dispersive forces (e.g., van der Waal forces and π-π interactions) between DOM molecules^48, 49^. A recent study also reported that the obvious overlap between the absorbance spectra of DOM and the emission spectra of polycyclic aromatic hydrocarbons can be attributed to the energy transfer from the polycyclic aromatic hydrocarbon to DOM^50^. DOM can also bind with trace contaminants^19, 51^ and heavy metals^32^ by forming molecular aggregates, altering the behavior, mobility and transport of these compounds. Nevertheless, the intermolecular energy transfer depends on the molecular complexity of the humic substance^52^. For example, the humic-like and protein-like components are expected to have different capacities to bind with trace contaminants or heavy metals. This needs further investigation in future studies.

## Conclusion

In this study, we investigated the roles of DOM in mediating the membrane filtration behavior of DFHC by revealing the interaction between the antibiotic-like components and humic-like components with the aid of EEM and PARAFAC modeling. The main findings in this work can be described as follows:

1. The membrane retention of DFHC in the absence of DOM was 42% and 35% using 20 kDa and 50 kDa ultrafiltration membranes, respectively. The presence of DOM, particularly HA, greatly increased the membrane retention rate of DFHC.
2. The co-occurrence of DFHC and DOM in a mixture led to significant changes in their EEM spectra, e.g., a more significant overlapping problem of EEM peaks and the blue or red shifting of some peaks.
3. The PARAFAC modeling successfully decomposed the EEM spectra into four independent components, including a DFHC component (C1) and three humic-like components belonging to the DOM (C2, C3 and C4). The fluorescence intensity of the C1 component (300 *μ*g/L) decreased by 25%, 5% and 3.5% when the concentrations of SRDOM, HA and SRDOM + HA reached approximately 12 mg/L, respectively. The DFHC component and humic-like components exhibited unidirectional interactions.
4. The fitted stability constant of the antibiotic-like component for SRDOM, HA and SRDOM + HA was in the order of HA > SRDOM + HA > SRDOM, which was consistent with the fluorescence-quenching efficiencies of DFCH. Overall, the fluorescence-quenching phenomena can well explain the DOM-regulated membrane rejection of the DFHC antibiotic.

## Electronic supplementary material

Supplementary data for this paper
