# Supplementary material for: DOM-mediated membrane retention of fluoroquinolone as revealed by fluorescence quenching properties
Source: Sci Rep. 2017 Jul 14;7:5372. doi: 10.1038/s41598-017-05635-z (PMC5511141; doi:10.1038/s41598-017-05635-z)
Supplement: Supplementary file 1 — Supplementary data for this paper [file 41598_2017_5635_MOESM1_ESM.pdf]

Supplementary Materials for:

DOM-mediated membrane retention of fluoroquinolone as revealed by fluorescence quenching properties

Shuang Liang [a], Li Lu [a], Fangang Meng [b]*

[a] Shandong Provincial Key Laboratory of Water Pollution Control and Resource Reuse, School of Environmental Science and Engineering, Shandong University, Jinan 250100, China

[b] School of Environmental Science and Engineering, Sun Yat-sen University, Guangzhou 510275, PR China

* **Corresponding author**

Fangang MENG, Ph. D.

Email: mengfg@mail.sysu.edu.cn

Tel: 86-20-39335060

Fax: 86-20-84110267

Table S1 Apparent molecular weight distribution for DOM at pH 7.0

	SRDOM (%)	HA (%)	SRDOM+HA (%)
> 100 kDa	17.10	48.95	55.82
10 ~ 100 kDa	8.89	27.61	12.14
3 ~ 10 kDa	15.41	6.40	18.34
< 3 kDa	58.60	17.05	13.70

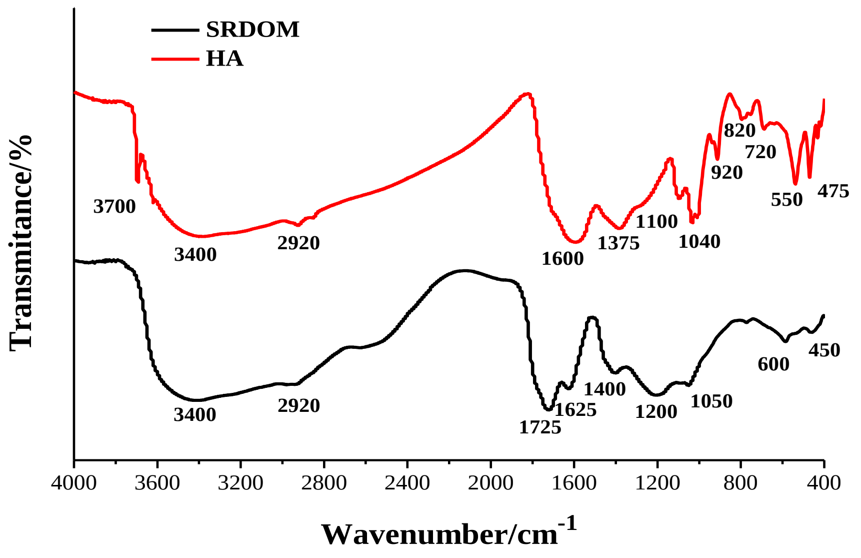

Figure S2 FTIR spectra of SRDOM and HA

Table S2 FTIR spectral features of SRDOM

Wavenumber (cm^{-1})	Band assignments	Reference
3400	O-H stretching of carboxyl, phenol and alcohol, stretching of the N-H bond , N-H bond of amino groups and the O-H bond of phenolic groups	Dalvi et al. 2000, Fu et al. 2016, Garrido Reyes et al. 2016, Xu et al. 2007
2920	aliphatic or alicyclic C-H stretching	Bai et al. 2015, Fu et al. 2016, Rodr ǵuez-Z úñiga et al. 2008, Xu et al. 2007
1725	C=O stretching of COOH or ketones, carboxylic C=O and unsaturated C=C stretching in humic acid	Bai et al. 2015, Fukushima et al. 2001, Liangliang et al. 2016, Xu et al. 2007
1625	C=O stretching of amide groups and aromatic substances, C=O bond in quinones substance, ketone C=O stretch and C=C in aromatic rings	Bo et al. 2012, Fu et al. 2016, Garrido Reyes et al. 2016, Wang et al. 2010, Wang et al. 2014, Xu et al. 2007
1400	C-O symmetric stretching of carboxyl moieties	
1200	C-O stretching and O-H deformation of carboxylic groups, C-O antisymmetric stretching of ether bonds	Bai et al. 2015, Fukushima et al. 2001, Jiaet al. 1977, Sobczyński et al. 2004)
1050	C-O group of polysaccharides	Guo et al. 2006, Liangliang et al. 2016, Xu et al. 2007
600	C-S bond	Dalvi et al. 2000, Maas 1972
450	shoulder peak of 600 cm^{-1} (C-S bond)	Dalvi et al. 2000

Table S3 FTIR spectral features of HA

Wavenumber (cm^{-1})	Band assignments	Reference
3700	phenolic O-H stretching	Fukushima et al. 2001
3400	stretching of carboxyl, phenol and alcohol and stretching of the N-H bond	Dalvi et al. 2000, Rodríguez and Núñez 2011, Xu et al. 2007
2920	aliphatic or alicyclic C-H stretching	Rodríguez-Zúñiga et al. 2008, Rodríguez and Núñez 2011, Xu et al. 2007
1600	C=O stretching of amide groups and aromatic substances, C=O bond in quinones substance and C=C stretch (alkenes and aromatic rings)	Guo et al. 2006, Rodríguez and Núñez 2011, Sobczyński et al. 2004, Xu et al. 2007
1375	alcohol or carboxylate groups (COO^-)	Augugliaro et al. 2012, Rodríguez and Núñez 2011, Sobczyński et al. 2004
1100	C-O symmetric stretching and the C-O group of polysaccharides	Guo et al. 2006, Xu et al. 2007
1040	C-O symmetric stretching, the C-O group of polysaccharides	Guo et al. 2006, Xu et al. 2007
820	C-C anti-symmetric ring stretching	Fukushima et al. 2001

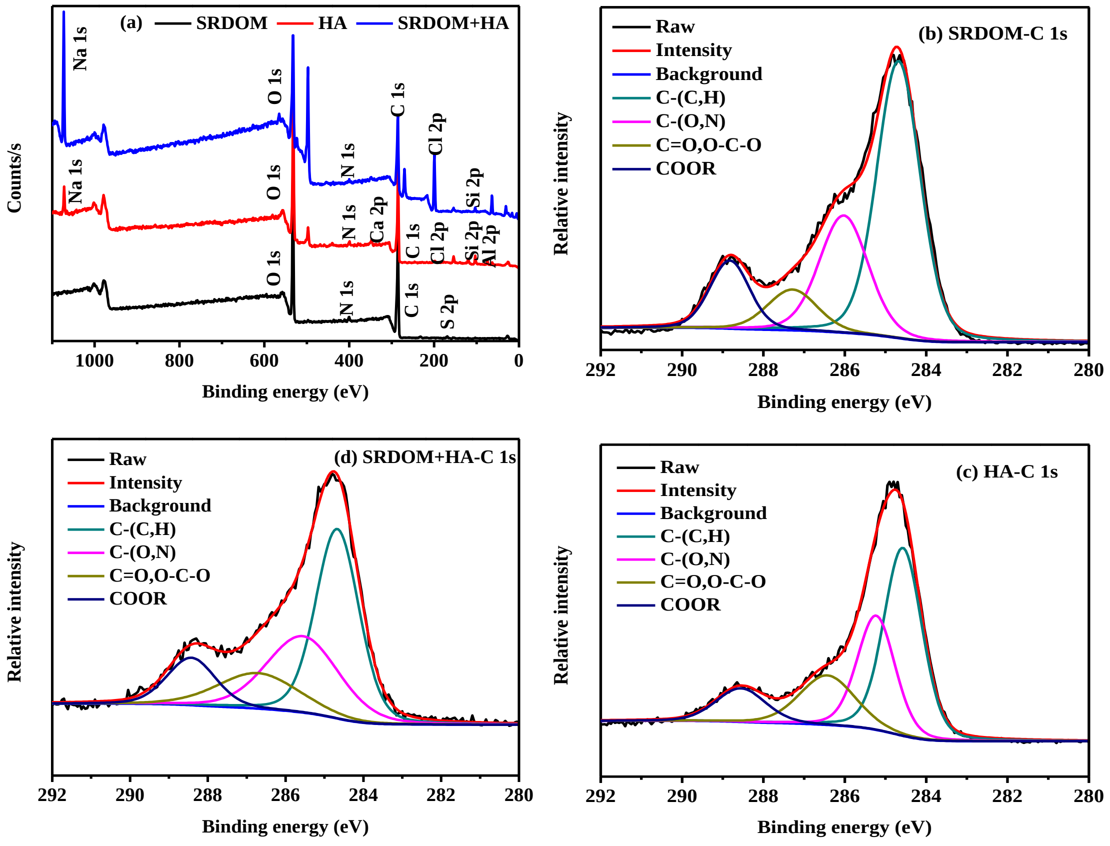

Figure S3 XPS spectra of elements in SRDOM, HA and SRDOM+HA (a) and high resolution C 1s spectra in SRDOM (b), HA(c) and SRDOM+HA (d)

Table S4 Atom fraction (%) of DOM determined by XPS

	Elemental composition (molar ratio with respect to total carbon)		Chemical functions (molar ratio with respect to total carbon)							
	O/C	N/C	284.6 eV	285.5 eV	287.6 eV	288.5 eV	531.3 eV	532.2 eV	399.9 eV	401.3 eV
			C-(C, H)	C-(O, N)	C=O, O-C-O	COOR	CO	C-OH, C-O-C	Nnonpr	Npr
SRDOM	1.3440	0.0466	0.535	0.255	0.091	0.120	0.000	1.204	0.030	0.008
HA	1.7637	0.0499	0.450	0.272	0.172	0.105	0.082	1.693	0.035	0.034
SRDOM+HA	1.9201	0.0467	0.434	0.292	0.152	0.122	0.046	1.363	0.020	0.001

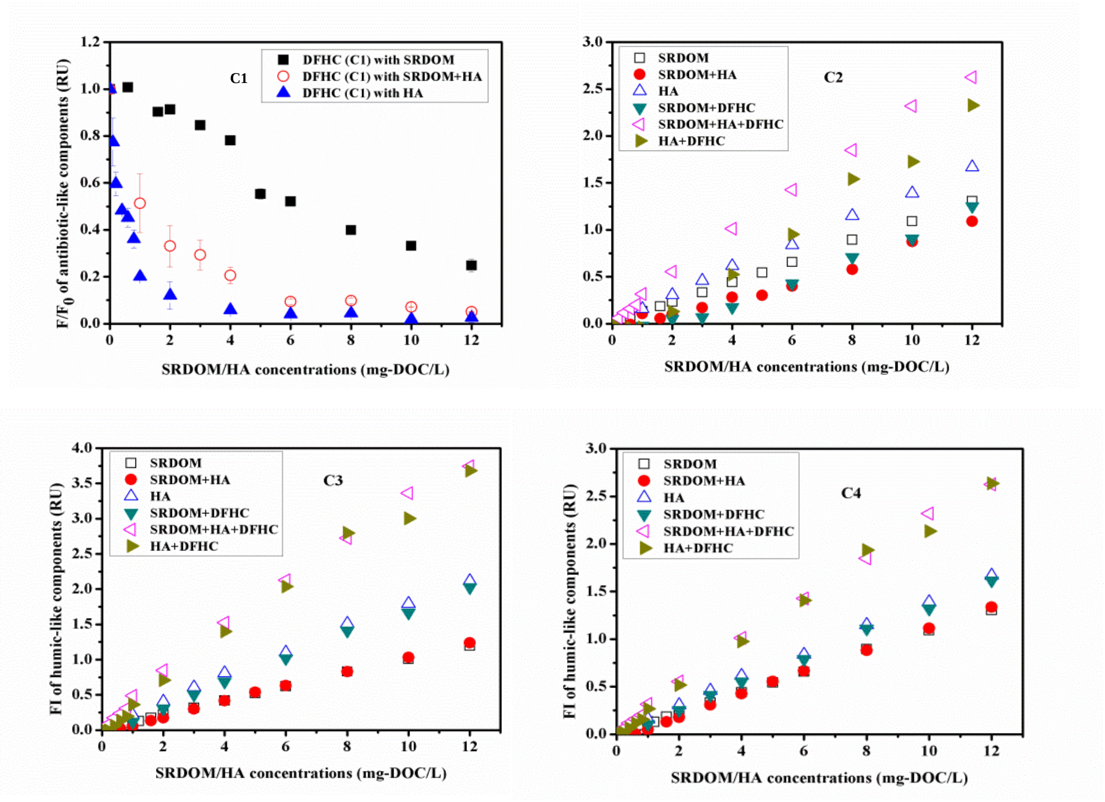

Figure S4 Representative plots of changes in fluorescence intensity of four components: (a) antibiotic-like component, (b) (c) (d) humic-like components

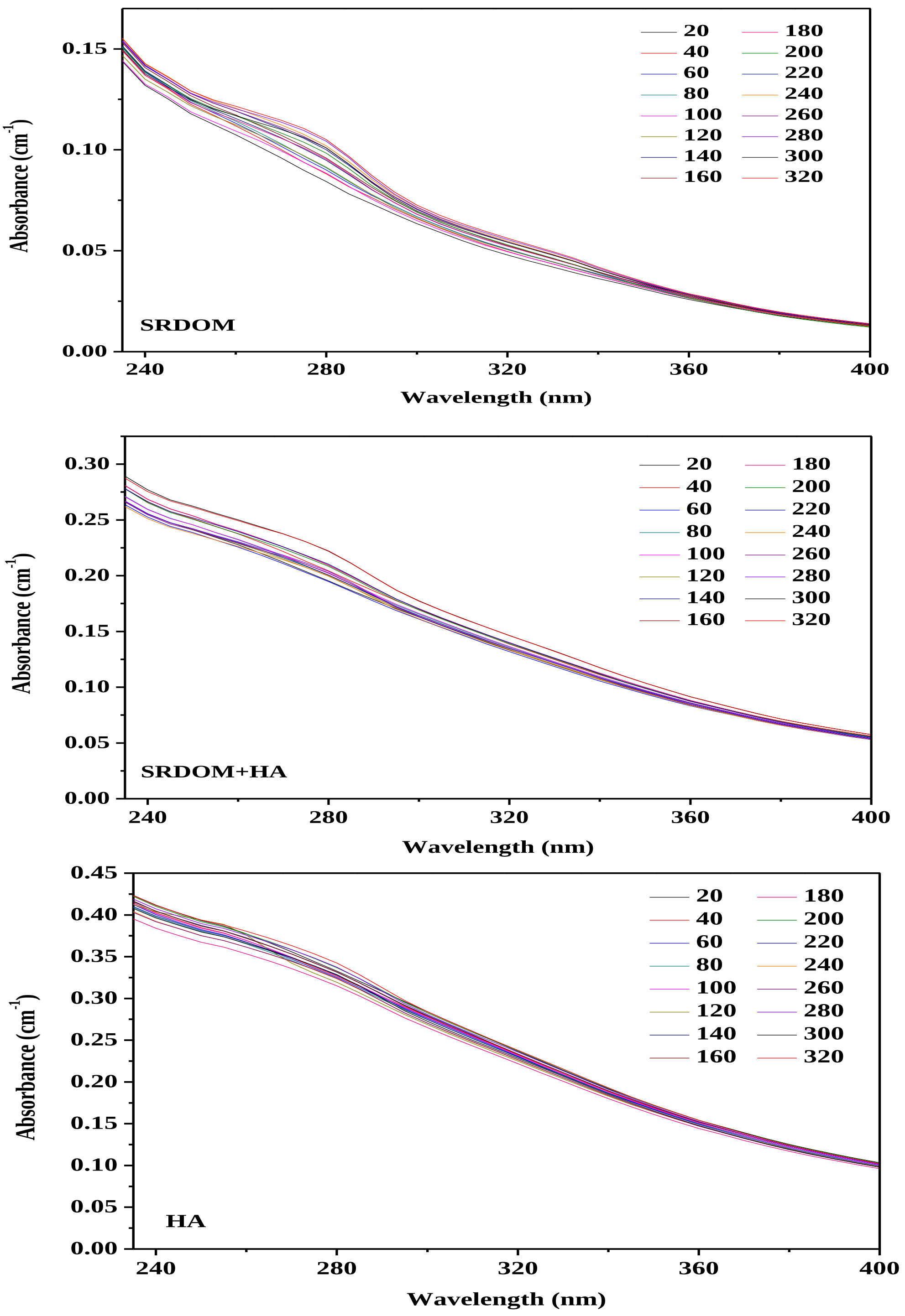

Figure S5 UV-vis spectra of SRDOM, SRDOM+HA and HA with the dosage of DFHC (0-320 μg/L)

References

Augugliaro, V., Bellardita, M., Loddo, V., Palmisano, G., Palmisano, L. and Yurdakal, S. (2012) Overview on oxidation mechanisms of organic compounds by TiO 2 in heterogeneous photocatalysis. Journal of Photochemistry & Photobiology C Photochemistry Reviews 13(3), 224–245.

Bai, Y., Wu, F., Xing, B., Meng, W., Shi, G., Ma, Y. and Giesy, J.P. (2015) Isolation and characterization of Chinese standard fulvic acid sub-fractions separated from forest soil by stepwise elution with pyrophosphate buffer. Scientific Reports 5.

Bo, P., Qiu, M., Min, W., Di, Z., Peng, H., Di, W. and Xing, B. (2012) The opposite impacts of Cu and Mg cations on dissolved organic matter-ofloxacin interaction. Environmental Pollution 161(1), 76-82.

Dalvi, A.G.I., Al-Rasheed, R. and Javeed, M.A. (2000) Studies on organic foulants in the seawater feed of reverse osmosis plants of SWCC ☆. Desalination 132(1-3), 217-232.

Fu, Q.L., He, J.Z., Blaney, L. and Zhou, D.M. (2016) Roxarsone binding to soil-derived dissolved organic matter: Insights from multi-spectroscopic techniques. Chemosphere 155, 225-233.

Fukushima, M., Tatsumi, K. and Nagao, S. (2001) Degradation characteristics of humic acid during photo-Fenton processes. Environmental Science & Technology 35(18), 3683-3690.

Garrido Reyes, T.I., Mendoza Crisosto, J.E. and Ascar Estay, M.L. (2016) CHARACTERIZATION OF THE DISSOLVED ORGANIC MATTER PRESENT IN THE WATER OF THE BÍO-BÍO RIVER, VIII REGION OF CHILE. Journal of the Chilean Chemical Society 61(2), 2890-2894.

Guo, Z., Ma, R. and Li, G. (2006) Degradation of phenol by nanomaterial TiO 2 in wastewater. Chemical Engineering Journal 119(1), 55-59.

Jia, H., Li, L., Fan, X., Liu, M., Deng, W. and Wang, C. (1977) Visible light photodegradation of phenanthrene catalyzed by Fe(III)-smectite: role of soil organic matter, Heyden.

Liangliang, WANG, Xiangjuan, KONG, Guangyi, Shuang, Qingliang, ZHAO and Fuyi (2016) Application of ultra-sonication, acid precipitation and membrane filtration for co-recovery of protein and humic acid from sewage sludge. Frontiers of Environmental Science & Engineering 10(2), 327-335.

Maas, J.J.V.D. (1972) Basic infrared spectroscopy [1972].

Rodríguez-Zúñiga, U.F., Milori, D.M., Da, S.W., Martin-Neto, L., Oliveira, L.C. and Rocha, J.C. (2008) Changes in optical properties caused by UV-irradiation of aquatic humic substances from the amazon river basin: seasonal variability evaluation. Environmental Science & Technology 42(6), 1948-1953.

Rodríguez, F.J. and Núñez, L.A. (2011) Characterization of aquatic humic substances. Water and

Environment Journal 7(2), 163-170.

Sobczyński, A., Duczmal, Ł. and Zmudziński, W. (2004) Phenol destruction by photocatalysis on TiO 2 : an attempt to solve the reaction mechanism. Journal of Molecular Catalysis A Chemical 213(2), 225-230.

Wang, G.S., Liao, C.H., Chen, H.W. and Yang, H.C. (2010) Characteristics of natural organic matter degradation in water by UV/H2O2 treatment. Environmental Technology 27(3), 277-287.

Wang, W., Wang, W., Fan, Q., Wang, Y., Qiao, Z. and Wang, X. (2014) Effects of UV radiation on humic acid coagulation characteristics in drinking water treatment processes. Chemical Engineering Journal 256(8), 137-143.

Xu, G., Li, F. and Wang, Q. (2007) Effect of humic acids on photodegradation of chloroacetanilide herbicides under UV irradiation. Journal of Environmental Science & Health Part B 42(2), 165-171.
